# The New England Quarterly Journal of Medicine and Surgery

**Published:** 1842-07

**Authors:** 


### Bibliographical Notices.

---

Art. II.—*The New England Quarterly Journal of Medicine and Surgery.* Vol. 1st, No. 1, for July, 1842. Boston, pp. 156.

Cunard's steamers and the "Western Railway" seem to have given an impetus to more things than commerce in Boston: a new-born excitement has taken hold of the profession. Hitherto, notwithstanding her advantages for such an enterprise, the only medical periodical published there has been a small, neat-looking, "one-masted" vessel, (we might have said one *mastered*) yclept the *Boston Medical and Surgical Journal,* under the guidance of that learned Theban and experienced navigator, Jerome Van Crowninshield Smith, M. D., of *ichthyological* memory, who is as punctual in his visitations as a bank notice, for which he deserves and receives due thanks. But here we have a new craft looming up in the distance, a regular "three-decker," we suppose it might be called, though not a vessel of war, which is to anchor and lay its cargo at our door every three months.

To drop such an incongruous metaphor, though, ere it has led us into error, if it has not already done it, and speak in landsman's phrase, as best becomes one who never snuffed a sea-breeze, we would simply say, that the above is the title of a new Quarterly, whose first number has been lying on our table some days, having with a sort of rail-road celerity reached us considerably in advance of its appointed time. It is edited by Charles E. Ware, M. D., and Samuel Parkman, M. D., names not unfamiliar to the reader, and is *backed* by some of the best pens in New England.

The first feeling we had, when, two months or more since, we copied a notice heralding the advent of this journal was one of surprise that something of the kind had not been undertaken long before; and we must still express the same feeling. It would be superfluous to say that there is plenty of talent and material in Boston for such a work, when some of our best writers, both surgical and medical, are or have been residents of that city. And as to support, the territory comprehended under the term New England, ought by all means to sustain two journals, even supposing their circulation to be confined to its limits, which is not the case with the weekly already mentioned, and of course will not be with the new quarterly.

Some of our exchanges—the New York Lancet for example—predict that it will be a failure on account of its *trimestrial* character, and express a doubt whether any thing can succeed save a cheap weekly journal. With due deference to them, we think differently. The rage (if such there be) for hebdomadals, will be short-lived, and work its own cure, to which nothing will tend more promptly or effectually than their multiplication. We do not deny them a measure of utility; they quickly disseminate a knowledge of the *novelties* occurring in the profession, in which respect they are attractive and peradventure useful; but from their very constitution they can do little more. For solid learning, for extensive research, for all, in short, that can give character and dignity to the profession, that will set its members to thinking and reflecting, and result in permanent improvement, a monthly or quarterly will be required. There are few readers, we opine, who do not look with far more interest and anticipated benefit for the American Journal of Medical Sciences, or some of the foreign quarterlies republished in this country (modesty forbids the mention of our *monthly* in such a connection), than for any of the weekly periodicals now existing among us. Boston has quick communication with the world beyond the waters, she has numerous and large hospitals, extensive libraries, a corps of liberal and enlightened medical men—wherefore, then, should she not possess a valuable and popular journal of the kind now offered to the public?

This number exhibits a handsome appearance—nothing flashy or dandyish—both inside and out. Its contents are of a diversified character, consisting of nine original papers on medical and surgical subjects, with reviews, bibliographical notices, scientific intelligence, and a variety of selected matter. The original papers are all of a valuable nature, such as will at once give a high standing to the work; one of them will be found in our pages for this month, and we shall take an early opportunity to introduce our readers to some others that we have marked for transfer. Under the head of scientific intelligence are extracts from reports made to the Boston Society for Medical Improvement, which contain much that is of interest; this is a feature that will be continued, we presume, in the future numbers. On the whole we augur very favorably of this periodical, and give it a cordial welcome. If any of our readers crave more food than is served up to them in our monthly dish, we commend the New England Quarterly to their favorable notice.

